# Ulcers

**Published:** 1890-10

**Authors:** A. McNeil

**Affiliations:** San Francisco; Bureau of Surgery, I. H. A.


### ULCERS.

A. McNeil, M. D., San Francisco.

(Bureau of Surgery, I. H. A.)

I will not weary you with definitions or descriptions of the varieties of ulcers, for these you already know. I will only give my views on the treatment of them. And in this I have nothing new to offer, only that laid down by Hahnemann. Any one who treats ulcers without keeping the psoric theory in his mind may perhaps get the credit of curing them, but he will in the end do more harm than good. In fact, he will relieve his patient of an annoyance, perhaps a painful one, and bring in something instead that will be much more distressing and perhaps fatal.

All kinds of external treatment, either operative or medicinal, are unnecessary and hurtful. No skin-grafting, stropping, astringent or, in fact, any medicinal salves or ointments should be permitted. When the indicated remedy has set the *vis medicatrix naturæ* at work she needs no external aid. And when the ulcer heals under this treatment it is because the psora, scrofula, or whatever name you may give it, is eradicated from the system.

As an evidence, I recall the first ulcer I treated. The patient was a woman, "fair, fat, and forty," who had all of her menstrual life been afflicted by an indolent ulcer on her leg *or* cardialgia. *Never both at once.* As long as the ulcer was open she enjoyed, with that exception, vigorous health. But always soon after it was healed, and it had been done many times by external treatment, she was attacked by the most violent and persistent colics I have ever witnessed. Is it reasonable to believe that she had two distinct diseases that never happened to attack her simultaneously? Or did she have only one disease which manifested itself in two distinct ways? I firmly believe the latter, and when nature was able to keep it on the surface, which she could do when not foiled by irrational interference, she had to suffer it to attack the nerves of the stomach.

It was necessary in making up the totality of the symptoms to include both those of the stomach and those immediately relating to the stomach. Calc-carb. covered both classes of symptoms, and given high relieved her of both for several years, when the ulcer again broke out and the same remedy produced a speedy and permanent cure.

Let me present a kindred case treated the other way. It was related by a physician who prefaced his treatment by saying, "I am a good homœopath." A child was brought to him with an aural polypus which had once been removed by the forceps. He did the same with caustics. Twice more at intervals it returned, to be again treated in the same way. It did not reappear after the fourth time, but instead indications of a long-continued abscess of the internal ear and the mastoid process. Cerebral symptoms followed, and death ensued. A post-mortem was held, and the astonishment of those present was great at seeing how life had continued with so great a necrosis of the bones of the ear and adjacent parts. My astonishment was equally great at witnessing the nonchalance with which he related how through his ignorance he had caused the death of a human being. "Where ignorance is bliss 'tis folly to be wise."

The vital question remains: How to cure ulcers? Hahnemann has already given us directions as to the mode of examining patients that has never been improved. Follow them carefully and conscientiously, and when that is done, as he has told us, the most difficult and important part is done. Do not venture to make an off-hand prescription unless it is one of those exceptional cases in which characteristic symptoms stand out plainly and it is not possible for another remedy to be the one. As aid in studying out the remedy there are no better ones than Bœnninghausen's *Pocket-book* and Gilchrist's *Surgical Diseases.* Use one, or better, both. With these and a fair amount of industry all ulcers may be cured, safely, pleasantly, and permanently.

Dr. J. T. Martin, of Woodland, California, reported a cure of necrosis of the tibia at the last meeting of the I. H. A. The bone lay bare and black, entirely denuded of periosteum to the extent of four and a half inches in length by over an inch in breadth. The most noted allopaths of the Sacramento Valley had tried and failed. He called me in consultation, and we agreed on Silicea, and in one year and a half, with eight or ten doses of that remedy high, the case was cured and the bone was covered by its normal coating, while the patient's general health was fully restored. The doctor carried out faithfully the plan of treatment agreed on. I might cite other cases, but I doubt not that all of you by strict Hahnemannian treatment have cured such cases.

